# Uroselective Alpha-1A Blockade Versus Surgical De-Obstruction: Differential Associations with Heart Rate Variability Restoration and Symptom Relief in Benign Prostatic Hyperplasia with Bladder Outlet Obstruction

**DOI:** 10.3390/life16040600

**Published:** 2026-04-04

**Authors:** Kuan-Yu Chen, Yu-Hui Huang, Yun-Sheng Chen, Min-Hsin Yang, Kai-Siang Chen, Chieh-Jui Chen, Cheng-Ju Ho, Chih-Kai Peng, Sung-Lang Chen

**Affiliations:** 1 Department of Urology, Chung Shan Medical University Hospital, Taichung 402, Taiwan; huracan1235@gmail.com (K.-Y.C.); barbarian06070136@gmail.com (M.-H.Y.); benaries108@hotmail.com (C.-J.H.); kenfreedom1903@gmail.com (C.-K.P.); 2 School of Medicine, Chung Shan Medical University, Taichung 402, Taiwan; yhhuang59@hotmail.com; 3 Department of Physical Medicine and Rehabilitation, Chung Shan Medical University Hospital, Taichung 402, Taiwan; 4 Department of Obstetrics and Gynecology, Changhua Christian Hospital, Changhua 500, Taiwan; gracelucky028@gmail.com; 5 The Center of Humanities and Society, Chia Nan University of Pharmacy & Science, Tainan 717, Taiwan; cks888@mail.cnu.edu.tw; 6 Graduate Institute of Business Administration, Fu Jen Catholic University, New Taipei 242, Taiwan; 7 School of Medicine, College of Medicine, Taipei Medical University, Taipei 110, Taiwan; chenjerry123@gmail.com

**Keywords:** heart rate variability, lower urinary tract symptoms, transurethral resection of the prostate, alpha blocker, benign prostatic hyperplasia

## Abstract

Background: Benign prostatic hyperplasia (BPH) can be associated with lower urinary tract symptoms (LUTS) and potential avlterations in autonomic nervous system function, as reflected by heart rate variability (HRV). This observational study was designed to generate hypotheses regarding the differential impacts of surgical de-obstruction versus uroselective pharmacological blockade on autonomic nervous system equilibrium, HRV restoration, and symptomatic outcomes in men with BPH and bladder outlet obstruction. Methods: Data from a prospective cohort of 242 men undergoing TURP and 210 men receiving tamsulosin were analyzed. HRV parameters (standard deviation of normal-to-normal intervals [SDNN], low-frequency/high-frequency [LF/HF] ratio, total power [TP], very low frequency [VLF]) and International Prostate Symptom Score (IPSS) was assessed at baseline and 12 weeks. Propensity score matching (PSM) was used to address baseline differences in age, prostate volume, IPSS, and baseline SDNN. Inter-group comparisons used ANCOVA with baseline as a covariate. Results: After TURP, SDNN increased by 14.70 ms (40%; 36.97 ± 22.80 to 51.67 ± 27.59 ms; *p* = 0.032; paired Cohen’s d = 0.58), LF/HF decreased by 0.90 (55%; 1.63 ± 1.60 to 0.73 ± 0.52; *p* = 0.028; d = −0.76), TP increased by 1303 ms^2^ (95%; 1367 ± 820 to 2670 ± 1420 ms^2^; *p* = 0.025; d = 1.12), and VLF increased by 810 ms^2^ (85%; 950 ± 560 to 1760 ± 980 ms^2^; *p* = 0.030; d = 1.01). For tamsulosin, SDNN increased by 6.73 ms (18%; 38.12 ± 12.50 to 44.85 ± 11.20 ms; *p* = 0.004; d = 0.57), LF/HF decreased by 0.16 (8%; 1.95 ± 0.65 to 1.79 ± 0.55; *p* = 0.012; d = −0.27), TP increased by 559 ms^2^ (39%; 1453 ± 620 to 2012 ± 580 ms^2^; *p* = 0.006; d = 0.93), and VLF increased by 355 ms^2^ (35%; 1020 ± 450 to 1375 ± 420 ms^2^; *p* = 0.010; d = 0.82). Secondary *p*-values (LF/HF, TP, VLF) were adjusted via the Benjamini–Hochberg method; adjusted *p* > 0.05 was used for some. Inter-group differences in changes were significant (ANCOVA *p* < 0.01; partial η^2^ = 0.12–0.22 for group factor). TURP was associated with greater IPSS reduction (−10.2 points; 18.5 ± 6.2 to 8.3 ± 4.1; *p* < 0.001) compared to tamsulosin (−5.3 points; 15.8 ± 5.6 to 10.5 ± 4.8; *p* < 0.001; d = −1.02; inter-group *p* < 0.001). PSM confirmed these associations with *p* < 0.01 for HRV changes. Change in SDNN was associated with IPSS improvement in multivariate regression (standardized β = −0.42, *p* < 0.01). Conclusions: In this observational study, TURP was associated with greater changes in HRV parameters and symptomatic improvement compared to tamsulosin. These findings are hypothesis-generating and require confirmation in long-term randomized trials.

## 1. Introduction

Benign prostatic hyperplasia (BPH) is a highly prevalent condition among aging men, representing one of the most common causes of lower urinary tract symptoms (LUTS). Epidemiological data indicate that the prevalence of BPH increases markedly with age, affecting approximately 8% of men in their 40 s, rising to 50% in their 60 s, and reaching up to 80% in those over 70 years [1,2,3,4,5]. Globally, the burden of BPH has grown substantially, with prevalent cases estimated at 112.5 million in 2021, reflecting a 122% increase since 1990, driven largely by population aging and growth [6]. This condition is increasingly recognized not merely as a localized urological issue but as potentially involving autonomic nervous system (ANS) alterations, particularly as reflected in heart rate variability (HRV), which has been associated with prostate size and symptom severity in some studies [7,8,9]. Studies have demonstrated that patients with BPH exhibit altered HRV parameters, including decreased total power (TP) and high-frequency (HF) components [10,11,12,13,14]. This may involve afferent signals from pelvic nerves, but such mechanisms remain speculative without direct measurement.

Treatment allocation to transurethral resection of the prostate (TURP) versus tamsulosin in real-world practice inherently reflects different disease stages, with more severe LUTS and larger prostates typically selected for surgery. Building upon our previous research [8], we reported that TURP was associated with significant improvements in LUTS and concurrent increases in global HRV indices at short-term follow-up. Specifically, surgical relief of obstruction was accompanied by increases in standard deviation of normal-to-normal intervals (SDNN) and TP and reductions in LF/HF (low-frequency/high-frequency) ratio, paralleling meaningful reductions in International Prostate Symptom Score (IPSS). However, studies on α-1A blocker treatment in BPH patients measuring their autonomic response are scarce and typically use non-selective α blockers [15], which may confound HRV assessments due to systemic cardiovascular effects. Standard pharmacological management often utilizes α-blockers as first-line therapy for symptomatic relief, but previous studies have highlighted risks associated with non-selective agents (e.g., doxazosin, terazosin), which may lead to blood pressure instability and increased incident heart failure (HR up to 1.08) [16,17,18]. To minimize potential cardiovascular confounding in HRV measurements, this study employs the uroselective agent tamsulosin (0.4 mg QD), which targets α-1A receptors with minimal systemic impact [19,20]. We therefore designed this observational study to generate hypotheses regarding the differential impacts of definitive surgical de-obstruction versus uroselective pharmacological blockade on ANS modulation, HRV restoration, and symptomatic outcomes, potentially informing personalized treatment strategies in an era of rising BPH prevalence.

## 2. Materials and Methods

This study was conducted in accordance with the Declaration of Helsinki and approved by the Institutional Review Board of Chung Shan Medical University Hospital (approval number: CS1-25122). Informed consent was obtained from all participants prior to enrollment.

### 2.1. Study Design and Participants

This was a prospective, non-randomized, observational cohort study reported in accordance with the STROBE guidelines, synthesizing data from two separate cohorts: one undergoing TURP and the other receiving tamsulosin therapy. As an observational study of routine clinical practice, the protocol was not registered on ClinicalTrials.gov. Participants were recruited from the Urology Department at Chung Shan Medical University Hospital between January 2023 and December 2025. Treatment allocation followed standard shared decision-making: patients with IPSS ≥ 15, prostate volume ≥ 50 mL, or intolerance/preference against long-term medication were offered TURP; others received tamsulosin. Prior medications (exclusively BPH-related agents such as α-blockers and 5-α-reductase inhibitors) were stopped at least 2 weeks before baseline for TURP patients. No washout was required in the tamsulosin cohort.

Inclusion criteria for both cohorts included: (1) male patients aged 50 years or older; (2) diagnosis of BPH with moderate-to-severe LUTS, defined as IPSS ≥ 8; (3) confirmed bladder outlet obstruction (BOO) via urodynamic studies (e.g., maximum flow rate < 15 mL/s and post-void residual volume > 50 mL or pressure-flow studies confirming); and (4) willingness to provide informed consent.

Exclusion criteria were: (1) primary cardiovascular disorders (e.g., uncontrolled hypertension, arrhythmia, or heart failure); (2) use of any medication with known direct effects on HRV (β-blockers, digoxin, antiarrhythmics, centrally acting sympatholytics); (3) history of prostate surgery or malignancy; (4) acute urinary tract infection or other urological conditions confounding symptoms; (5) neurological disorders affecting bladder function; and (6) inability to comply with follow-up assessments.

TURP Cohort: A total of 242 men (mean age 67.4 ± 6.8 years) underwent standard monopolar TURP.

Tamsulosin Cohort: A total of 210 men (mean age 69.8 ± 7.5 years) were treated with Harnalidge^®^ OCAS^®^ prolonged release tablets at 0.4 mg QD (Astellas Pharma Inc., Tokyo, Japan).

Comorbidities: Treated hypertension (TURP: 45%, tamsulosin: 48%; *p* = 0.62), diabetes (22% vs. 25%; *p* = 0.48), BMI (24.5 ± 3.2 vs. 24.8 ± 3.4 kg/m^2^; *p* = 0.41), smoking history (30% vs. 28%; *p* = 0.71), and subclinical cardiovascular risk factors not significantly different (*p* > 0.05). There was no coronary artery disease in either group due to exclusions. Diabetes duration was not recorded but was controlled in regression.

### 2.2. Assessments

HRV was measured using 24 h Holter monitoring (SEER Light, GE Healthcare, Chicago, IL, USA) at baseline and exactly 12 weeks post-intervention, standardized to start at 9 a.m. with normal activity instructions. Parameters: SDNN (primary), LF/HF, TP, and VLF (secondary; VLF included for long-term regulatory insights, despite interpretive challenges [21]). Mean excluded beats: 2.8% in TURP vs. 2.5% in tamsulosin (*p* = 0.45). Artifact percentage was <5%, ectopy excluded; no sleep stage or respiratory correction was applied. Data were analyzed with the MARS software version 9.0 (GE Healthcare). IPSS, including sub-scores and QoL, was assessed at baseline and 12 weeks [22].

### 2.3. Statistical Analysis

Data were presented as means ± standard deviation. Paired *t*-tests were used for within-group changes. Inter-group comparisons were performed using ANCOVA with baseline values as covariates for 12-week outcomes (dependent variable: post-intervention value). SDNN was predefined as the primary HRV endpoint; others were secondary (Benjamini–Hochberg’s correction for secondary *p*-values). Pearson’s correlations (r) were used. Multivariate linear regression was used for ΔSDNN predicting IPSS, controlling for age and prostate volume (standardized β, adjusted R^2^, VIF). PSM (1:1, caliper 0.2) was performed on age, prostate volume, IPSS (because these were the strongest baseline imbalances and primary outcome correlates), and baseline SDNN (primary overall HRV measure; other HRV parameters were highly correlated with SDNN, r > 0.7, to avoid overmatching); SMD < 0.1 post-matching. While PSM balanced measured covariates (SMD < 0.1), it cannot adjust for unmeasured confounders such as physical activity, sleep quality, duration of BPH symptoms, alcohol intake, autonomic neuropathy or diabetes duration, which were not recorded and represent an acknowledged limitation of PSM for causal inference. SPSS 27.0 was used. Sample size provided >80% power (alpha = 0.05) to detect medium effect sizes (Cohen’s d = 0.5) in primary HRV differences (SDNN) based on prior data [8].

## 3. Results

Baseline characteristics of the unmatched cohorts are summarized in Table 1.

### 3.1. Autonomic Nervous System Modulation

TURP: SDNN increased by 14.70 ms (95% CI: 1.3–28.1; *p* = 0.032; paired Cohen’s d = 0.58), LF/HF decreased by 0.90 (*p* = 0.028; adjusted *p* = 0.042; d = −0.76), TP increased by 1303 ms^2^ (*p* = 0.025; adjusted *p* = 0.042; d = 1.12), and VLF increased by 810 ms^2^ (*p* = 0.030; adjusted *p* = 0.042; d = 1.01) (Table 2; VLF in Appendix A). Tamsulosin: SDNN increased by 6.73 ms (*p* = 0.004; d = 0.57), LF/HF decreased by 0.16 (*p* = 0.012; adjusted *p* = 0.018; d = −0.27), TP increased by 559 ms^2^ (*p* = 0.006; adjusted *p* = 0.018; d = 0.93), and VLF increased by 355 ms^2^ (*p* = 0.010; adjusted *p* = 0.018; d = 0.82) (Table 3; VLF in Appendix A). Inter-group comparisons via ANCOVA showed significant differences (*p* < 0.01 for SDNN, LF/HF, TP, VLF; partial η^2^ = 0.15 for SDNN, 0.12 for LF/HF, 0.22 for TP, 0.18 for VLF). Baseline TP and VLF were lower in TURP (*p* < 0.05).

### 3.2. Symptomatic Efficacy

TURP: IPSS reduction by 10.2 points (*p* < 0.001; d = −1.94). Tamsulosin: IPSS reduction by 5.3 points (*p* < 0.001; d = −1.02); inter-group ANCOVA *p* < 0.001 (partial η^2^ = 0.25) (Table 4). Voiding sub-scores improved more in TURP (Δ-7.1; adjusted *p* < 0.01), correlated with HRV changes (r = −0.42, *p* < 0.05). Correlations were moderate-to-strong in TURP (r = 0.45–0.55, *p* ≤ 0.01) and were weaker in tamsulosin (r = 0.28–0.35, *p* < 0.05 for some, *p* = 0.056 for others) (Table 5). Regression: ΔSDNN was associated with ΔIPSS (β = −0.42, *p* < 0.01; R^2^ = 0.28; VIF < 1.5) and ΔQoL (β = −0.38, *p* < 0.05; R^2^ = 0.25).

### 3.3. Propensity Score Matched Analysis

There were 210 matched pairs; baselines were balanced (e.g., IPSS 16.2 ± 5.8 vs. 15.8 ± 5.6, *p* = 0.78; SMD < 0.1) (Table 6). PSM confirmed TURP associations with greater ΔSDNN (+13.8 vs. +6.73 ms, *p* < 0.01), ΔLF/HF (−0.85 vs. −0.16, *p* < 0.01), ΔTP (+1240 vs. +559 ms^2^, *p* < 0.01), ΔVLF (+770 vs. +355 ms^2^, *p* < 0.01), and IPSS Δ-9.8 vs. Δ-5.3 (*p* < 0.001). Correlations were similar in matched subsets. Subgroup for <50 mL prostates was attenuated but consistent (Table 2).

Note: Extremely high correlations (e.g., r = 0.9558 for LF and IPSS) were cited from the prior literature [8]; current data show moderate r values. Scatterplots for key correlations are provided in Appendix A.

### 3.4. Safety Outcomes

No major surgical complications occurred (transfusion rate 1.2%, re-operation 0.8%). In the tamsulosin group, adverse events were mild (dizziness 7.6%, retrograde ejaculation 12.4%); no cardiovascular events were recorded in either arm.

## 4. Discussion

This observational study observed associations between TURP and greater changes in HRV parameters and symptomatic relief compared to tamsulosin in men with BPH. While both interventions were associated with improvements, the differences may reflect baseline severity or intervention mechanisms, though causality cannot be inferred due to the non-randomized design.

### 4.1. The BPH-ANS Association

Converging evidence suggests that ANS regulates urological inflammatory and immune reflex, and HRV has been proposed to monitor human inflammatory processes [23]. Our findings reinforce the concept that BPH is not merely a mechanical obstruction but a condition rooted in central sympathetic overactivity. Clinical evidence demonstrates a highly significant positive correlation between the LF HRV component (a marker of sympathetic tone) and both IPSS (r = 0.9558, *p* < 0.001) and prostate volume (r = 0.9421, *p* < 0.001). Chronic BOO creates a state of sustained physiological stress, likely via afferent signals from the pelvic nerves that maintain a “fight-or-flight” state [7,9].

Baseline HRV differences—lower LF/HF but depressed SDNN, TP, and VLF in TURP cohort—may suggest varying autonomic profiles by disease severity, where severe cases show reduced overall variability. This pattern has parallels in chronic conditions like heart failure or chronic stress [24,25], but direct extrapolation is cautious without autonomic nerve activity or catecholamine measures. The observational data support an association between BOO severity and HRV depression. A notable observation is the lower baseline LF/HF ratio in the TURP group (1.63 vs. 1.95) despite more severe symptoms. While evidence is lacking, this may reflect autonomic adaptation in advanced disease, contrasting with milder cases. This is speculative and requires direct autonomic measures for validation.

### 4.2. Mechanical Relief Versus Pharmacological Blockade

TURP was associated with larger changes in HRV parameters, which may reflect alterations in autonomic regulation; however, HRV metrics (particularly the LF/HF ratio) cannot establish specific sympathovagal mechanisms [21]. By physically resecting the obstructive tissue, TURP deactivates the chronic stress on the detrusor muscle, potentially resetting the sympathovagal balance toward parasympathetic dominance [26].

In contrast, α-blockers function via receptor antagonism to relax smooth muscle, providing rapid but gradual relief without removing the structural drivers of neural stress [27]. These patterns may be consistent with different stages of BPH pathophysiology: early hyperactivity in moderate disease vs. chronic blunting and fatigue in severe cases. The gradual BOO relief in α-blockers (achievable within 3 months for milder profiles) facilitates adaptive stabilization, while TURP’s abrupt mechanical release enables deeper recovery, particularly in “high responders” (older patients ≥ 65 years or prostates ≥ 50 mL, showing ~60% greater HRV gains) [8]. The mean IPSS reduction observed with tamsulosin in our cohort (−5.3 points) is considerably lower than the −8-point reductions typically reported in large European multicenter trials [28]. Similarly, quality of life (QoL) improvements appeared also smaller than documented in those cohorts. This discrepancy may be attributable to our stricter exclusion criteria and the exclusive use of a purely uroselective agent without systemic vasodilatory effects. These design features likely produced a more conservative symptom improvement profile in a real-world Asian population with well-controlled comorbidities, highlighting potential ethnic or population-specific differences in treatment response.

Our synthesis shows that pharmacological therapy tends to move the system toward a baseline equilibrium (LF/HF ~1.79), similar with healthy volunteers [29], rather than boosting parasympathetic tone to the levels seen post-surgery, though LF/HF is a frequency-domain index with controversial interpretation [21]. Differences may arise from baseline disparities or selection bias, mitigated but not eliminated by PSM. Furthermore, medications cannot address non-adrenergic stressors like endothelin-1, which contribute to the 30–35% non-responder rate in pharmacological BPH therapy [14,16].

### 4.3. Disparity in HRV Improvements: SDNN and LF/HF

The notable disparity in HRV improvements between groups warrants discussion. TURP elicited more profound changes (ΔSDNN +14.70 ms vs. +6.73 ms; ΔLF/HF −0.90 vs. −0.16; *p* < 0.01 for both inter-group deltas), reflecting greater vagotropic recovery. This can be attributed to baseline differences and intervention mechanisms. At baseline, the TURP cohort exhibited more impaired overall autonomic tone (lower SDNN: 36.97 ms vs. 38.12 ms; lower TP: 1367 ms^2^ vs. 1453 ms^2^; lower VLF: 950 ms^2^ vs. 1020 ms^2^) but less pronounced sympathetic skew (lower LF/HF: 1.63 vs. 1.95), suggestive of autonomic blunting and fatigue in advanced BPH, where prolonged sympathetic overdrive leads to receptor desensitization and blunted responses, supported by the lower baseline TP and VLF indicating reduced overall variability. This pattern may arise from the TURP group’s higher symptom severity (mean IPSS 18.5 vs. 15.8), indicating more chronic BOO, which could induce autonomic blunting and fatigue, reducing the LF/HF ratio despite overall reduced variability (low SDNN). In contrast, the tamsulosin group’s milder baseline symptoms suggest earlier-stage BPH with active sympathetic hyperactivity (higher LF/HF) but relatively preserved autonomic variability (higher SDNN, TP, VLF). These baseline disparities, likely due to selection bias where severe cases opt for surgery, explain why TURP yields greater improvements: starting from a more exhausted state allows for substantial “reset” via mechanical relief, while tamsulosin normalizes milder imbalances without profound shifts.

Correlations between IPSS/QoL improvements and HRV changes (Table 5) were modest-to-moderate associations in the TURP group (e.g., ΔIPSS vs. ΔSDNN: r = −0.55, *p* < 0.001; ΔQoL vs. ΔSDNN: r = −0.52, *p* < 0.001), suggesting that more robust autonomic restoration contributes to greater symptomatic relief, potentially via reduced neural stress signaling. In the tamsulosin group, correlations were weaker but still significant for IPSS (e.g., ΔIPSS vs. ΔSDNN: r = −0.35, *p* < 0.05), indicating a link between modest autonomic normalization and symptom alleviation, though less pronounced than in TURP, consistent with the pharmacological approach’s limited impact on underlying neural drivers. This underscores TURP’s role in severe cases for holistic recovery, while tamsulosin suits milder profiles for stabilization. Although our previous work [8] reported very high correlations (r = 0.9558), the current independent cohort shows only moderate associations (r = 0.45–0.55), consistent with typical clinical physiology findings and highlighting the exploratory nature of earlier observations. PSM analysis (Table 6) and matched correlations confirmed these patterns, with TURP maintaining stronger associations even after balancing baselines, indicating robustness beyond selection bias.

### 4.4. Theoretical Safety of Selective Blockade and Cardiovascular Implications

Large-scale analyses, including Medicare databases, have shown elevated hazard ratios for major adverse cardiovascular events (MACE) and new-onset heart failure (HR up to 1.08) with non-selective α blockers, such as doxazosin and terazosin, compared to selective agents or alternatives like 5-α reductase inhibitors [16,19,20]. By targeting α-1B and α-1D receptors in the vasculature in addition to α-1A in the prostate, they often lead to significant vasodilation, resulting in orthostatic hypotension, dizziness, and syncope, particularly in older patients or those with comorbidities. This can increase fall risk and exacerbate symptoms in individuals with autonomic instability. Furthermore, the blockade of α-2 receptors by some non-selective agents removes a negative feedback loop on norepinephrine release, leading to compensatory surges in catecholamines, which can cause tachycardia, palpitations, and tremulousness. Tamsulosin was chosen for its α-1A selectivity, minimizing vascular effects and risks seen with non-selective agents (e.g., HR 1.08 for heart failure) [1,6,18]. Selective blockers are theoretically safer due to their receptor specificity and are safer for comorbidities, though side effects like floppy iris syndrome or retrograde ejaculation [30,31] may impact QoL.

TURP’s larger HRV changes hypothesize potential benefits via autonomic stability, but no endpoints were measured, and findings are speculative without long-term data. Furthermore, the observed greater HRV shifts with TURP, such as more pronounced increases in SDNN and TP and reductions in LF/HF, suggest a stronger autonomic modulation compared to tamsulosin. This could imply that surgical intervention, by providing definitive mechanical relief of BOO, achieves a more substantial restoration of autonomic balance toward parasympathetic dominance [8,15]. Previous HRV studies in BPH used non-selective α-blockers with systemic cardiovascular effects [15]. By employing the uroselective agent tamsulosin, the present work isolates prostate-specific autonomic modulation while minimizing confounding, thereby addressing a key limitation of the earlier literature. The comparative HRV improvements with TURP highlight the potential for surgery to confer broader systemic benefits, though this remains hypothesis-generating and requires confirmation [1,2,31].

### 4.5. Limitations

Several limitations warrant consideration. First, there was a baseline disparity in symptom severity between the two cohorts; the TURP group presented with a significantly higher total IPSS (18.5 ± 6.2) compared to the α-blocker group (15.8 ± 5.6). This discrepancy likely reflects a selection bias inherent in real-world clinical practice, where more symptomatic patients or those intolerant to pharmacological side effects such as orthostatic hypotension choose surgical intervention to achieve more substantial relief. Although PSM mitigated this, residual confounding may persist. Second, this research utilized a non-randomized, observational design based on the synthesis of two separate prospective cohorts. Because patients were not randomly assigned to treatments, unmeasured confounders—such as differing levels of baseline physical activity or lifestyle factors—could have influenced the HRV outcomes [32]. Additionally, the sample size remained relatively small (*n* = 242 for the TURP group and *n* = 210 for the medication group), which may limit the generalizability of the findings and the power of the subgroup analyses. Third, this study strictly evaluated the gold-standard TURP procedure [33,34] and did not enroll patients undergoing newer BPH surgical techniques, such as laser prostatectomy, Urolift, or Rezum. Recent minimally invasive surgical options have shown superior preservation of sexual function—a clinical marker of sympathetic autonomic integrity—in recent systematic review and meta-analyses [35], suggesting potentially reduced autonomic disruption compared with conventional TURP. As surgical innovation continues to evolve toward minimally invasive options, the autonomic impact of these newer modalities remains a critical area for future investigation. Fourth, the placebo effect of surgery may partially inflate the IPSS delta in the TURP group, as it is well-documented that surgical patients report higher subjective improvements due to the “theatrical” nature of surgery compared to daily pill intake [36]; however, while IPSS (subjective) might be influenced by placebo, HRV (objective/autonomic) is an involuntary physiological marker, making the autonomic findings more reliable than the symptom scores alone. Finally, the 12-week endpoint captures early changes; longer-term data (6–12 months) would be required to confirm durability, as LUTS and HRV parameters may continue to improve beyond this period. While our data show stable trends (e.g., no regression in matched correlations), the possibility of transient influences from surgical healing on 3-month HRV cannot be ruled out, emphasizing the need for longer follow-up to confirm durability.

## 5. Conclusions

In this observational study, TURP was associated with greater improvements in HRV parameters and symptoms than tamsulosin, potentially due to mechanical BOO relief. However, because of the non-randomized design, causality cannot be inferred, and the findings remain hypothesis-generating; randomized trials are needed for long-term outcomes, and personalized strategies, possibly using HRV as a biomarker, are required.

## Figures and Tables

**Table 1:** Baseline characteristics of the cohorts (unmatched).

Characteristic	TURP Cohort (*n* = 242)	Tamsulosin Cohort (*n* = 210)	*p*-Value
Age (years, mean ± SD)	67.4 ± 6.8	69.8 ± 7.5	0.002
Prostate Volume (mL, mean ± SD)	58.2 ± 20.1	52.4 ± 18.6	0.010
Baseline IPSS (mean ± SD)	18.5 ± 6.2	15.8 ± 5.6	<0.001
Baseline Qmax (mL/s, mean ± SD)	9.8 ± 3.4	11.2 ± 3.1	0.004
Treated Hypertension (%)	45	48	0.620
Diabetes (%)	22	25	0.480
BMI (kg/m^2^, mean ± SD)	24.5 ± 3.2	24.8 ± 3.4	0.410
Smoking History (%)	30	28	0.710
Baseline SDNN (ms, mean ± SD)	36.97 ± 22.80	38.12 ± 12.50	0.520
Baseline LF/HF (mean ± SD)	1.63 ± 1.60	1.95 ± 0.65	0.030
Baseline TP (ms^2^, mean ± SD)	1367 ± 820	1453 ± 620	0.042
Baseline VLF (ms^2^, mean ± SD)	950 ± 560	1020 ± 450	0.030

Note: *p*-values from unpaired *t*-tests or chi-square as appropriate. No CAD in either group due to exclusions.

**Table 2:** Pre- and post-TURP HRV changes (unmatched cohort).

Parameter	Baseline (Mean ± SD)	12 Weeks (Mean ± SD)	Delta (Mean)	*p*-Value (Paired *t*-Test)	Adjusted *p*-Value (Benjamini–Hochberg)
SDNN (ms)	36.97 ± 22.80	51.67 ± 27.59	+14.70	0.032	N/A (Primary)
LF/HF	1.63 ± 1.60	0.73 ± 0.52	−0.90	0.028	0.042
TP (ms^2^)	1367 ± 820	2670 ± 1420	+1303	0.025	0.042
VLF (ms^2^)	950 ± 560	1760 ± 980	+810	0.030	0.042

**Table 3:** Pre- and post-tamsulosin HRV changes (unmatched cohort).

Parameter	Baseline (Mean ± SD)	12 Weeks (Mean ± SD)	Delta (Mean)	*p*-Value (Paired *t*-Test)	Adjusted *p*-Value (Benjamini–Hochberg)
SDNN (ms)	38.12 ± 12.50	44.85 ± 11.20	+6.73	0.004	N/A (Primary)
LF/HF	1.95 ± 0.65	1.79 ± 0.55	−0.16	0.012	0.018
TP (ms^2^)	1453 ± 620	2012 ± 580	+559	0.006	0.018
VLF (ms^2^)	1020 ± 450	1375 ± 420	+355	0.010	0.018

**Table 4:** Comparative outcomes for key parameters (unmatched cohort, ANCOVA inter-group comparisons).

Parameter	TURP Delta (Mean)	Tamsulosin Delta (Mean)	ANCOVA *p*-Value	Partial η^2^ (Group Factor)	Cohen’s d (Inter-Group)
SDNN (ms)	+14.70	+6.73	<0.001	0.15	0.62
LF/HF	−0.90	−0.16	<0.001	0.12	−0.85
TP (ms^2^)	+1303	+559	<0.001	0.22	0.98
VLF (ms^2^)	+810	+355	<0.001	0.18	0.92
Total IPSS (points)	−10.2	−5.3	<0.001	0.25	−1.15
Voiding Sub-score	−7.1	−3.8	<0.001	0.20	−1.02
QoL Index	−2.5	−1.4	0.002	0.14	−0.78

**Table 5:** Correlations between changes in IPSS/QoL and HRV parameters.

Correlation Pair	TURP Group (r, *p*-Value)	Tamsulosin Group (r, *p*-Value)
ΔIPSS vs. ΔSDNN	−0.55, *p* < 0.001	−0.35, *p* = 0.028
ΔIPSS vs. ΔLF/HF	0.45, *p* = 0.002	0.28, *p* = 0.056
ΔQoL vs. ΔSDNN	−0.52, *p* < 0.001	−0.32, *p* = 0.032
ΔQoL vs. ΔTP	0.50, *p* = 0.001	0.30, *p* = 0.041

**Table 6:** Standardized mean differences (SMD) post-PSM for key variables.

Variable	TURP (Mean ± SD)	Tamsulosin (Mean ± SD)	SMD
Age (years)	68.2 ± 7.1	68.5 ± 7.3	0.04
Prostate Volume (mL)	55.4 ± 18.2	54.8 ± 17.9	0.03
Baseline IPSS	16.2 ± 5.8	15.8 ± 5.6	0.07
Baseline SDNN (ms)	37.5 ± 20.1	37.8 ± 19.8	0.02

## Data Availability

The original contributions presented in this study are included in the article. Further inquiries can be directed to the corresponding author.

## Abbreviations

The following abbreviations are used in this manuscript:

BPH	Benign Prostatic Hyperplasia
LUTS	Lower Urinary Tract Symptoms
ANS	Autonomic Nervous System
HRV	Heart Rate Variability
TURP	Transurethral Resection of Prostate
SDNN	Standard Deviation of Normal-to-Normal Intervals
IPSS	International Prostate Symptom Score
BOO	Bladder Outlet Obstruction
QoL	Quality of Life
MACE	Major Adverse Cardiovascular Events

## Appendix A

**Table A1:** Pre- and Post-Intervention VLF Changes (Unmatched Cohort).

Parameter Group	Baseline (Mean ± SD)	12 Weeks (Mean ± SD)	Delta (Mean)	*p*-Value (Paired *t*-Test)	Adjusted *p*-Value (Benjamini–Hochberg)	Cohen’s d
VLF (ms^2^) TURP	950 ± 560	1760 ± 980	+810	0.030	0.042	1.01
VLF (ms^2^) Tamsulosin	1020 ± 450	1375 ± 420	+355	0.010	0.018	0.82

Note: VLF included for exploratory long-term regulatory insights, despite interpretive limitations [30]. Baseline VLF lower in TURP (*p* < 0.05). Inter-group ANCOVA *p* < 0.01; partial η^2^ = 0.18.

**Table A2:** Pre- and Post-Intervention VLF Changes (PSM Matched Cohort).

Parameter Group	Baseline (Mean ± SD)	12 Weeks (Mean ± SD)	Delta (Mean)	*p*-Value (Paired *t*-Test)	Adjusted *p*-Value (Benjamini–Hochberg)	Cohen’s d
VLF (ms^2^) TURP	960 ± 550	1730 ± 950	+770	0.028	0.042	0.95
VLF (ms^2^) Tamsulosin	1015 ± 445	1370 ± 415	+355	0.012	0.018	0.81

Note: *n* = 210 matched pairs. Inter-group ANCOVA *p* < 0.01; partial η^2^ = 0.17. Baselines balanced post-PSM (SMD < 0.1).
